# Changing Meal Sequence Affects Glucose Excursions in Gestational Diabetes Mellitus

**DOI:** 10.1155/2022/7083106

**Published:** 2022-07-21

**Authors:** Guangjin Yong, Qian Jing, Qing Yao, Kechun Yang, Xinhua Ye

**Affiliations:** Department of Endocrinology, The Affiliated Changzhou No. 2 People's Hospital of Nanjing Medical University, Changzhou 213003, China

## Abstract

Studies on nutrient sequences during meals suggest that consuming carbohydrates last lowers postprandial glucose excursions more than consuming carbohydrates first. However, this phenomenon has not been studied in gestational diabetes mellitus (GDM). Ten women with GDM consumed the same caloric foods in different sequences over five successive days: (A) dish first, followed by carbohydrate and soup last; (B) carbohydrate first, followed by dish and soup last; (C) soup first, followed by dish and carbohydrate last; (D) three meals a day ad libitum; and (E) six meals a day as ad libitum. Continuous glucose monitoring (CGM) was used to assess diurnal glycemia. Decreases in mean glucose levels and the largest glucose levels in A were similar to group C. The peak glucose of breakfast and lunch in group B was more significant than in groups A and C. The B meal pattern showed more marked glycemic excursions than groups A and C. Increasing the number of meals reduced the peak glucose level and the glycemic excursions with the same total calories. Changing meal sequences or increasing the number of meals may reduce glycemic excursions in GDM. Our trial was registered retrospectively and the trial registration number is ChiCTR2200057044.

## 1. Introduction

Gestational diabetes mellitus (GDM) is hyperglycemia diagnosed during pregnancy, characterized by glucose levels below those considered diagnostic of overt diabetes outside of pregnancy [1]. Maternal overweight and obesity, later age at childbearing, reduced physical activity, previous history of GDM, family history of type 2 diabetes mellitus, and ethnicity are significant risk factors [2, 3]. The hyperglycemia of GDM affects maternal birth outcomes, increases adverse neonatal events, and is associated with long-term health problems in affected mothers and their offspring [4–8]. Diet and exercise are the first-line therapy for GDM, and insulin is used when normoglycemia is not achieved as well as some oral hypoglycemic drugs, principally metformin and glimepiride which are just recommended in some countries [9–11].

Medical nutrition therapy is the cornerstone of the treatment of GDM, which has been shown to improve glycemic control and should be initiated shortly after diagnosis [3, 12]. A meal sequence in which dietary fibers are eaten first, followed by the protein and fat courses and finally carbohydrates, originates from the traditional Japanese “Kaiseki” cuisine [13]. Crossover studies found that changing the meal sequence markedly improves postprandial glucose excursion, gastric emptying, and incretin secretion in individuals with and without diabetes [14–18]. Consuming vegetables before carbohydrates also reduces whole-day glucose excursions in type 2 diabetes [19]. However, no such studies have been conducted to determine whether changing the sequences of meals throughout the day has similar glycemic control effects in GDM. Continuous glucose monitoring (CGM) systems are sensors that automatically monitor blood glucose levels throughout the day and night by measuring glucose concentration in interstitial fluid [20]. This tool aids the understanding of blood glucose characteristics and has been recognized as an ideal monitoring system for glycemic control of diabetic patients [21, 22].

The present study was aimed at measuring the acute effects of various meal sequences and increasing the number of meals on glucose excursions in GDM by CGM with the same caloric load. We hypothesized that the most significant glycemic excursions would occur when carbohydrates were consumed first, and increasing the number of meals would reduce glycemic excursions.

## 2. Materials and Methods

### 2.1. Participants

Subjects were recruited from the outpatient clinic of Changzhou Second People's Hospital, China, from July 2020 to July 2021. The inclusion criteria for the GDM group were according to the recommendations of the International Association of Diabetes and Pregnancy Study Groups [23] (fasting glucose of ≥5.1 mmol/L, or ≥10.0 mmol/L after 1 h, or ≥8.5 mmol/L after 2 h in oral glucose tolerance test with 75 g of glucose). We excluded women with fasting plasma glucose ≥ 7.0 mmol/L, preexisting diabetes, multiple pregnancies, or other metabolic diseases (e.g., hyperthyroidism and liver or kidney dysfunctions). All participants provided written informed consent.

### 2.2. Study Design

In this randomized crossover trial, all participants consumed isocaloric meals for five consecutive days (Table 1). Participants were trained on premeal glucose collection and time recording before the test started. They were required to record glucose levels before meals and sleep, and they recorded the times of glucose collection and taking the first bite of each meal during the test. Breakfast was served at 7:30–8:30, lunch at 11:30–12:30, and dinner at 17:30–18:30.

The CGM system consists of a disposable Enlite sensor (Medtronic MiniMed, Northridge, USA) and a reusable recorder to measure glucose concentrations in interstitial fluid every 5 mins, generating 288 measurements per day. They were placed on the women's left arm before the test started. Meal sequences were divided into five groups. (A) dish first, followed by carbohydrate and soup last; (B) carbohydrate first, followed by dish and soup last; (C) soup first, followed by dish and carbohydrate last; (D) three meals a day ad libitum; and (E) six meals a day ad libitum and the extra meals were served at 9:30, 14:30, and 19:30. The total calories were the same in the five days. The sequences of A, B, and C were three meals a day, and the time of each meal was controlled for more than 30 min without intervals after each food intake. Groups D and E did not restrict the time and sequence of each meal, only the number of meals. Participants were closely monitored by having a video with the researcher at mealtime to ensure they could finish the meal as requested. A cell phone was required to be constantly carried except when bathing and sleeping. The total steps in a day's activity were limited to no more than 8,000 steps, recorded through the mobile phone applications. Strenuous exercises were avoided for 3 hours after meals during the test. A dietitian formulated the dietary composition of the test days according to the height, weight, and eating habits during pregnancy. The hospital cafeteria prepared the test meals according to the prepared recipes and delivered them to the subjects before eating.

### 2.3. Data Collection

Baseline data were collected for all participants. After the test, the sensor was removed from the participant, and the recorder was connected to the computer software to download the retrospective glucose data. We measured the following: (1) glucose level before meals, defined as the blood glucose value before taking the first bite of a meal; (2) peak glucose level, defined as the highest glucose level measured during the 3-hour postprandial period and time to peak glucose was defined as the point in time when the highest glucose level was measured; (3) mean glucose levels, calculated from the glucose levels measured by CGMs; (4) the daily largest and smallest glucose levels, provided by CGMs; and (5) area under the curve, calculated as the area under the glucose curve during the 3-hour postprandial period. The glucose excursions were assessed by the following parameters from the CGMs: standard deviation, the largest amplitude of glycemic excursions, the mean amplitude of glycemic excursions, and coefficient variation.

### 2.4. Statistical Analysis

Demographics for participants and results were expressed as mean ± standard deviation unless otherwise stated. All analyses were performed using SPSS software (version 26.0). Normally distributed continuous outcome parameters between the three groups were tested using a one-way analysis of variance. In case of abnormal distribution, the Kruskal-Wallis *H* test was used. Comparison between the groups D and E was performed using the paired Student's *t*-test. A general linear model was used to analyze blood glucose levels at various time points after meals. Differences with *P* < 0.05 were considered statistically significant.

## 3. Results

### 3.1. Flow and Participant Characteristics

**Figure 1:**
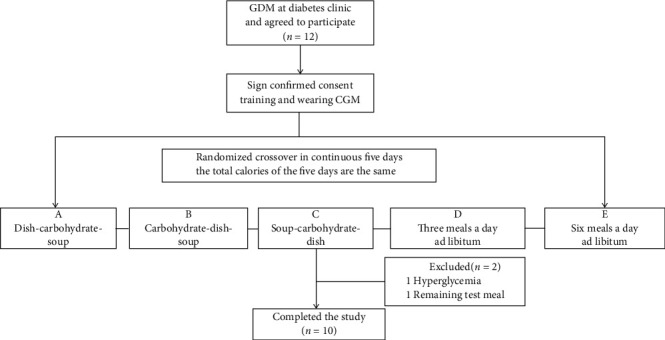
Patient selection and randomization.

Figure 1 displays a flowchart of participant selection and randomization. Twelve women started the test, and two were excluded. One participant was excluded because fasting glucose levels were greater than 7 mmol/L for two consecutive days, and another was excluded because of insufficient intake of the test meal. The remaining ten participants completed the study. Baseline characteristics are displayed in Table 2.

### 3.2. Glucose Levels

There were no significant differences in glucose levels at midnight across the five days. Baseline glucose concentrations of breakfast, lunch, and dinner were similar in the three conditions (Table 3). Compared with groups A and C, there were significantly higher peak glucose levels after breakfast and lunch in group B (*P* < 0.05); however, there were no differences in the time to peak after meals. The mean glucose level, the largest glucose level, and the area under the curve of the 3-hour postprandial were significantly lower in groups A and C food intake sequences than in group B (*P* < 0.05). We defined the subject's first bite as *T* = 0 and recorded glucose levels at 15 min intervals from 0 to 180 min of each meal; we found that the glucose level increased significantly between 45 and 75 minutes. The 3-hour postprandial glucose level is shown in Supplemental Figure 1.

### 3.3. Glucose Excursions

The standard deviation, mean amplitude of glycemic excursions, the largest amplitude of glycemic excursions, and coefficient variation were significantly lower in groups A and C than in B group (*P* < 0.05); however, there were no differences between groups A and C (Table 3).

### 3.4. Increasing the Number of Meals

Increasing the number of meals decreased the standard deviation, mean amplitude of glycemic excursions, the largest amplitude of glycemic excursions, coefficient variation, and peak glucose level (*P* < 0.05), but not the mean glucose level and the lowest glucose level (Table 4). Supplemental Figure 2 shows the diurnal glucose levels in groups D and E.

## 4. Discussion

Postprandial glucose is influenced by various factors such as the quality and quantity of carbohydrates, the size of a meal, and the presence and percentage of macronutrients (i.e., fat, protein, and the amount and type of dietary fiber). Gastric emptying, hormonal secretion, hepatic insulin extraction, and endogenous glucose production also influence postprandial glucose levels [24–26]. The quality and quantity of carbohydrates are the primary predictors of glycemic response, and low glycemic index (GI) or low glycemic load (GL) dietary patterns improve glycemic control in type 2 diabetes [27, 28]. The GI of food rich in carbohydrates estimates how quickly carbohydrates break down during digestion and how rapidly they are absorbed into the circulation [29]. Several factors determine the GI of a food, including the type of carbohydrate, the content of protein, fat, the quantity and type of fiber, food particle size, and pH [30]. The GL can provide information on peak glucose and the duration of glycemia when ingesting a specific amount of carbohydrate-rich food, providing an accurate picture of a food's real-life impact on postprandial glycemia. The sequence of macronutrients ingested and meal timing can also influence postprandial glucose. In 2017, the first Dietary Guidelines for Type 2 Diabetes of China included the “vegetable-meat-staple food” meal order in their core recommendations. Changing meal sequence does not affect total calories and is straightforward, making it more acceptable than the previous methods of restricting calorie intake.

Glucose excursion (defined by the amplitude, frequency, and duration of glycemic fluctuations around mean blood glucose) encompasses diurnal hyperglycemic peaks and hypoglycemic troughs [31]. HbA1c reflects long-term glucose control by identifying states of sustained hyperglycemia in the preceding 2–3 months; however, hypoglycemic episodes, short-term glucose fluctuations, and transient hyperglycemia do not significantly change HbA1c [32]. Glucose excursions are becoming a target for diabetes management, with postprandial glucose, glycated hemoglobin, and fasting glucose [33]. The literature suggests that glucose excursion is an independent risk factor for diabetes complications by affecting oxidative stress pathways, impairing endothelial cell function, and exacerbating chronic inflammatory states [34, 35].

In this study, we found that changing the sequence of food intake or increasing the number of meals affected glucose levels and glucose excursion when total calorie intake was fixed. Group B, where carbohydrates were consumed first, followed by dish and soup, had the highest maximum and mean glucose values. Peak glucose after breakfast and lunch in group B was higher than in the other two groups, but peak glucose levels after dinner and time to peak after meals did not differ among the three test days. Many factors can influence postprandial glucose, including the circadian clock, meal timing, and activity [36–38]. The peak postprandial glucose after dinner in group B was higher than in groups A and C after dinner; however, the difference did not reach statistical significance, and we believe this finding may be related to the following factors. Most participants were full-time housewives who tended to rest after breakfast and lunch with fewer activities during the day but may increase their activity after dinner because of walking with families. Although strenuous exercises were avoided for 3 hours after meals during the test, activities such as walking were allowed, and only total daily activity was limited to 8,000 steps. We did not detail the activity for the 3 hours after each meal and did not analyze the correlation between activity and peak glucose levels. Although the total daily calorie was the same, the macronutrient content of each test meal a day could not be kept identical. The GI and GL of foods combined in a meal were different. Bao et al. showed that GL was the strongest predictor of glycemia after mixed meals, explaining 58% of the observed variation; however, their metabolic responses were studied only at breakfast time. At the same time, human hormone secretion varies throughout the day, which may also affect postprandial glucose. Therefore, to improve hyperglycemic spikes in the postprandial state, it is essential not to overlook the role of multiple factors. We observed that the glucose level increased significantly between 45 and 90 minutes by recording glucose levels at 15 min intervals after meals. The sequence of consuming carbohydrates first gave a significantly higher glucose excursion than the other two groups, suggesting that this eating behavior should be avoided in GDM. Having more meals frequently with less food is one of the most common dietary guidelines for people with GDM, while the data on glucose excursion are limited. Increasing the number of meals by spreading the total calories over more meals to reduce the calories of each meal would lower maximum glucose levels and overall glucose excursion.

The study's strengths include the randomized controlled design, the use of CGM with a frequent assessment of maternal glucose concentrations across the day, and without time intervals between each food intake which was consistent with daily eating habits. We just required the patients to slow their eating speed, making the total meal time more than 30 minutes. The study also has limitations, including the small sample size, no washout day, and no mechanism study. Further studies with larger samples are needed to determine its long-term effectiveness and possible mechanisms.

In conclusion, this study showed that changing the sequence of food intake and increasing the number of meals is a simple and economical strategy to attenuate glycemic excursions in GDM and is expected to reduce the incidence of complications and improve the quality of life in mothers and offspring.

## Figures and Tables

**Table 1:** Test meal composition of 1800 kcal.

ABCD Breakfast			Lunch		Dinner	
Carbohydrates	Steamed bread	50 g	Black rice	200 g	Black rice	200 g
Soup	Skim milk	250 mL	Seaweed soup	150 mL	Seaweed soup	150 mL
Dish	Boiled eggs	60 g	Shredded chicken	20 g	Firm tofu	100 g
			Green bean sprout	100 g	Miniature cabbage	150 g
			Chinese flowering cabbage	100 g	Oil (olive)	15 g
			Oil (olive)	15 g	Shrimp meat	100 g
			Beef fillet	80 g	Dressing (ready)	20 g
			Dressing (ready)	20 g		
E Breakfast			Lunch		Dinner	
Carbohydrates	Steamed bread	25 g	Black rice	100 g	Black rice	100 g
Soup	Skim milk	250 mL	Seaweed soup	150 mL	Seaweed soup	150 mL
Dish	Boiled eggs	60 g	Shredded chicken	20 g	Firm tofu	100 g
			Green bean sprout	100 g	Miniature cabbage	150 g
			Chinese flowering cabbage	100 g	Oil (olive)	15 g
			Oil (olive)	15 g	Shrimp meat	100 g
			Seaweed soup	150 g	Dressing (ready)	20 g
			Dressing (ready)	20 g		
Morning tea			Afternoon tea		Supper	
Steamed bread		25 g	Boiled corn	150 g	Boiled corn	150 g
Total (kcal/d)			Protein (g)	Fat (g)	Carbohydrate (g)	
1867.1			92.6	71.9	212.4	

**Table 2:** Baseline characteristics (*n* = 10) of study population.

	Mean ± SD
Age (y)	30.10 ± 3.19
Gestational weeks (w)	26.70 ± 4.20
Height (cm)	161.80 ± 10.83
Weight (kg)	79.45 ± 18.32
BMI (kg/m^2^)	30.12 ± 2.41
Fasting glucose (mmol/L)	5.14 ± 0.66
Postprandial glucose 1 h (mmol/L)	6.67 ± 2.04
Postprandial glucose 2 h (mmol/L)	6.77 ± 2.23
HbA1c (%)	5.20 ± 0.50
Systolic blood pressure (mmHg)	114.60 ± 5.30
Diastolic blood pressure (mmHg)	76.80 ± 10.00

Abbreviations: BMI: body mass index; HbA1c: hemoglobin A1c. Each value indicates the mean ± SD.

**Table 3:** Glycemic outcome parameters of sequence ABC.

	A Dish-carbohydrate-soup	B Carbohydrate-dish-soup	C Soup-dish-carbohydrate	*P*
Glucose level before breakfast (mmol/L)	5.09 ± 0.68	5.45 ± 0.81	5.10 ± 0.52	0.395
Glucose level before lunch (mmol/L)	4.43 ± 0.39	4.73 ± 0.66	4.65 ± 0.85	0.583
Glucose level before dinner (mmol/L)	4.78 ± 0.38	4.90 ± 0.43	4.72 ± 0.35	0.581
Peak glucose of breakfast (mmol/L)	7.85 ± 1.06^∗^	8.58 ± 1.40	7.27 ± 1.16^∗^	0.017
Peak glucose of lunch (mmol/L)	6.43 ± 1.07^∗^	8.00 ± 1.65	6.77 ± 1.23^∗^	0.026
Peak glucose of dinner (mmol/L)	7.31 ± 1.56	8.71 ± 2.57	7.26 ± 2.21	0.133
Time to peak glucose of breakfast (min)	66.00 ± 11.74	55.75 ± 18.18	64.00 ± 14.68	0.419
Time to peak glucose of lunch (min)	62.50 ± 19.19	61.50 ± 15.10	75.00 ± 17.16	0.168
Time to peak glucose of dinner (min)	72.00 ± 20.44	71.50 ± 12.70	82.00 ± 25.08	0.430
Mean glucose level (mmol/L)	5.37 ± 0.57^∗^	6.18 ± 0.75	5.51 ± 0.56^∗^	0.020
Largest glucose level (mmol/L)	7.58 ± 1.40^∗^	9.47 ± 2.02	8.50 ± 1.49^∗^	0.014
Smallest glucose level (mmol/L)	4.06 ± 0.55	4.06 ± 0.45	4.11 ± 0.63	0.973
Standard deviation (mmol/L)	0.85 ± 0.24^∗^	1.27 ± 0.50	0.91 ± 0.40^∗^	0.048
Largest amplitude of glycemic excursions (mmol/L)	3.52 ± 1.04^∗^	5.41 ± 1.76	4.39 ± 1.49^∗^	0.013
Mean amplitude of glycemic excursions (mmol/L)	2.30 ± 1.07^∗^	3.93 ± 1.49	2.74 ± 1.27^∗^	0.015
Coefficient variation (%)	15.72 ± 2.91^∗^	21.72 ± 5.86	16.145 ± 5.59^∗^	0.020
AUC_0–3h_ of breakfast (mmol/L)	1105.63 ± 138.22^∗^	1389.20 ± 299.55	1127.70 ± 166.71^∗^	0.007
AUC_0–3h_ of lunch (mmol/L)	989.06 ± 125.01^∗^	1247.83 ± 203.60	1066.24 ± 194.77^∗^	0.009
AUC_0–3h_ of dinner (mmol/L)	1103.50 ± 165.60^∗^	1386.80 ± 241.30	1100.41 ± 238.96^∗^	0.005

Data are presented as mean ± SD; ^∗^*P* < 0.05 compared with sequence B; 1-factor ANOVA and Kruskal-Wallis test were used and least-significant difference (LSD) was used for the post hoc test; AUC: the area under the glucose curve during the 3-hour postprandial period.

**Table 4:** Characteristics of glucose excursion by different numbers of meals.

	D Three meals a day	E Six meals a day	*P*
Largest glucose level (mmol/L)	8.65 ± 1.52	7.44 ± 0.82	0.002
Smallest glucose level (mmol/L)	4.17 ± 0.50	4.26 ± 0.60	0.703
Mean glucose level (mmol/L)	5.33 ± 0.56	5.38 ± 0.48	0.598
Standard deviation (mmol/L)	1.03 ± 0.49	0.73 ± 0.32	0.002
Largest amplitude of glycemic excursions (mmol/L)	4.82 ± 2.06	3.34 ± 1.28	0.003
Mean amplitude of glycemic excursions (mmol/L)	2.30 ± 1.37	1.94 ± 1.22	<0.001
Coefficient variation (%)	19.12 ± 7.73	13.41 ± 4.98	0.001

All values are means ± SD. Data is analyzed by paired Student's *t*-test. *P* < 0.05 was considered statistically significant.

## Data Availability

The data that support the findings of this study are openly available at http://doi.org/[doi].

## Supplementary Materials

Supplementary 1 Supplement figure 1: the postprandial glucose levels at 15 min intervals from 0 to 180 mins after each meal. ^∗^*P* < 0.05 compared with B and A sequences. ^#^*P* < 0.05 compared with B and C sequences.

Supplementary 2 Supplement figure 2: the mean (±SEM) diurnal glucose levels following three meals a day (gray dots) and six meals a day (black dots). Average mealtimes are represented by the black arrows.
