# Supplementary material for: Changing Meal Sequence Affects Glucose Excursions in Gestational Diabetes Mellitus
Source: J Diabetes Res. 2022 Jul 21;2022:7083106. doi: 10.1155/2022/7083106 (PMC9338731; doi:10.1155/2022/7083106)
Supplement: Supplementary 1 — Supplement figure 1: the postprandial glucose levels at 15 min intervals from 0 to 180 mins after each meal. ∗P < 0.05 compared with B and A sequences. #P < 0.05 compared with B and C sequences. [file 7083106.f1.pdf]

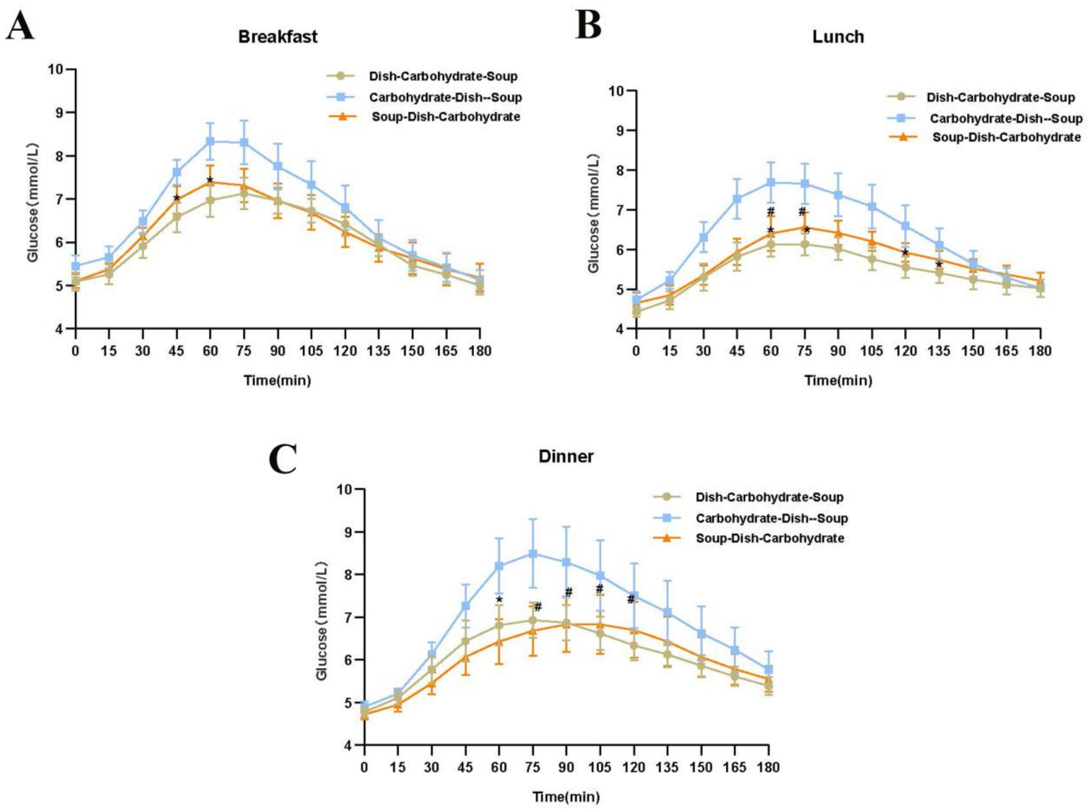

Supplement figure 1： The postprandial glucose levels at 15 min intervals from 0-180 mins after each meal.* *P*<0.05 compared with B and A sequence. # *P*<0.05 compared with B and C sequence.
