# Supplementary material for: Changing Meal Sequence Affects Glucose Excursions in Gestational Diabetes Mellitus
Source: J Diabetes Res. 2022 Jul 21;2022:7083106. doi: 10.1155/2022/7083106 (PMC9338731; doi:10.1155/2022/7083106)
Supplement: Supplementary 2 — Supplement figure 2: the mean (±SEM) diurnal glucose levels following three meals a day (gray dots) and six meals a day (black dots). Average mealtimes are represented by the black arrows. [file 7083106.f2.pdf]

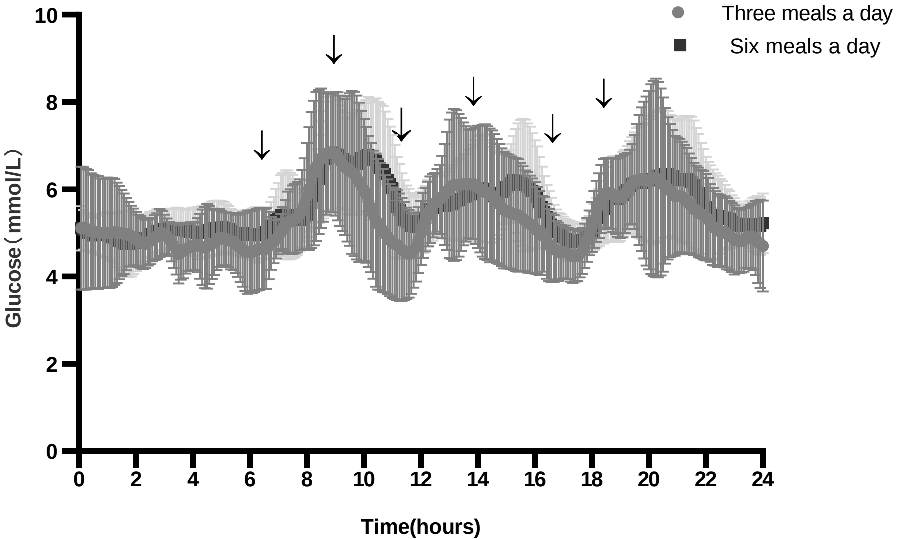

Supplementary Figure 2:Mean (±SEM) diurnal glucose levels following three meals a day (gray dots) and six meals a day (black dots).Average mealtimes are represented by the black arrows.
